# Effects of intermittent theta burst to the left dorsolateral prefrontal cortex on brain volumes and neurometabolites in people with alcohol use disorder: a preliminary investigation

**DOI:** 10.3389/fnhum.2025.1613993

**Published:** 2025-07-22

**Authors:** Timothy C. Durazzo, Lauren H. Beauregard, Meng Gu, Eric P. Kraybill, Brian D. P. Joseff, Amy A. Herrold, Keith Humphreys, M. Windy McNerney, Brian Knutson, Claudia B. Padula

**Affiliations:** ^1^Sierra-Pacific Mental Illness Research and Education Clinical Centers, Veterans Affairs Palo Alto Healthcare System, Palo Alto, CA, United States; ^2^Department of Psychiatry and Behavioral Sciences, Stanford University School of Medicine, Stanford, CA, United States; ^3^Department of Radiology, Stanford University School of Medicine, Stanford, CA, United States; ^4^Edward Hines Jr., VA Hospital, Hines, IL, United States; ^5^Department of Psychiatry and Behavioral Sciences, Feinberg School of Medicine, Northwestern University, Chicago, IL, United States; ^6^Center for Innovation to Implementation, Veterans Affairs Palo Alto Healthcare System, Menlo Park, CA, United States; ^7^Department of Psychology, Stanford University, Stanford, CA, United States

**Keywords:** alcohol use disorder, intermittent theta burst stimulation (iTBS), randomized clinical trial, brain volumes, brain metabolites, longitudinal

## Abstract

**Background:**

Randomized, placebo-controlled clinical trials (RCTs) employing repetitive transcranial magnetic stimulation (TMS) in the treatment of alcohol use disorder (AUD) have shown promising results. However, the mechanism(s) by which TMS produces improved outcomes in AUD are not established. The goal of these secondary analyses was to assess for longitudinal changes in brain volumes and neurometabolites in the left dorsolateral prefrontal cortex (DLPFC)—the stimulation site—across two published RCTs evaluating intermittent theta burst (iTBS) as an adjunct treatment for AUD.

**Materials and methods:**

Veterans with AUD (*n* = 44) were recruited from a residential treatment program at the VA Palo Alto Health Care System. Participants in this report were in RCTs evaluating the efficacy of iTBS for the treatment of AUD. Across studies, 21 participants were randomized to active iTBS and 23 to sham iTBS (2–3 iTBS active or sham sessions/day), delivered over approximately 2 weeks. Bilateral volumes of the rostral and caudal middle frontal and superior frontal gyri left DLPFC neurometabolites were quantitated pre- and post-iTBS sessions.

**Results:**

Over the 2-week assessment interval, significant volume increases were observed, collapsed across groups, in the bilateral rostral and caudal middle frontal and superior frontal gyri, as well as in the left DLPFC choline-containing compounds. No group (active vs. sham) × time (2-week assessment interval) interactions were apparent for any measure. Preliminary simple effect tests for volumes indicated that the active group demonstrated significant increases in the bilateral rostral and caudal middle frontal and superior frontal gyri, while the sham group only showed significantly increased left superior frontal volume. Preliminary simple effect tests for metabolites indicated that the active group had significant increases in left DLPFC choline-containing and creatine-containing compounds, and sham showed no significant metabolite changes. In the active group, a higher number of iTBS pulses delivered at the target treatment level was significantly associated with greater increases in left DLPFC n-acetylaspartate, glutamate, and gamma-aminobutyric acid.

**Conclusion:**

This study provided novel preliminary indications that iTBS promoted adaptive structural and neurometabolic changes in the left DLPFC site of stimulation in those with AUD. Replication of these findings in a larger sample and examination of other neuroimaging-based markers of TMS-induced neurobiological changes are critical to informing modifications of existing TMS protocols to maximize durable positive treatment outcomes in those with AUD.

## 1 Introduction

Several double-blind, randomized, placebo-controlled clinical trials (RCTs) have evaluated the efficacy of repetitive transcranial magnetic stimulation (TMS) as a treatment for alcohol use disorder (AUD). Collectively, TMS RCTs employing 10 or more sessions delivering active/real 10 or 20 Hz intermittent theta burst (iTBS) or continuous theta burst stimulation to the dorsolateral prefrontal cortex (DLPFC), medial anterior frontal cortex, or anterior cingulate cortex reported reduced alcohol craving and consumption, immediately post-active TMS, relative to sham stimulation (Padula et al., 2024) (see Padula et al., 2022; Mehta et al., 2024 for relevant reviews). Across coil types, pulse parameters, and diagnosed conditions, TMS is posited to promote adaptive behavioral change via neuroplastic modifications of cortical–cortical and/or cortical–subcortical circuits associated with the neocortical or paralimbic node stimulated (George et al., 2007; Philip et al., 2020; Antonelli et al., 2021). More specifically, the therapeutic benefits of TMS, across conditions, are broadly ascribed to changes in synaptic plasticity (Jannati et al., 2023), which are largely related to brain structural and biochemical changes in neuronal and, potentially, glial tissue. However, the actual structural and biochemical changes induced by TMS in humans with AUD, as well as the neurobiological mechanisms promoting the associated improved clinical outcomes, are not fully explicated (Kirkovski et al., 2023; Cole et al., 2024; Mehta et al., 2024; Padula et al., 2024).

The field has frequently utilized resting state functional connectivity (rsFC) as a neurobiological marker of TMS target engagement and/or treatment response (Fox et al., 2012; Beynel et al., 2020; Cole et al., 2024). While rsFC may serve as a potentially accurate brain-based marker of TMS target engagement and treatment response, particularly for treatment-resistant major depressive disorder (MDD) (Long et al., 2024), it is unclear if rsFC has equivalent ability to predict TMS treatment response in AUD. In RCTs that conducted pre- and post-TMS rsFC neuroimaging in AUD, treatment results depended on the region stimulated (e.g., Jansen et al., 2015; Harel et al., 2022). Additionally, rsFC does not provide information on potential TMS-related changes in the integrity of the brain parenchyma of the cortical node or associated circuit(s) stimulated. Therefore, a better understanding of the underlying brain structural and neurometabolic factors associated with TMS-induced neuroplastic changes in AUD will improve understanding of the neurobiological effects of TMS in humans, as well as the ability to identify those likely to respond to a particular type of TMS stimulation (e.g., excitatory versus inhibitory protocols).

In those with AUD, it is well established that regional brain morphology and neurometabolites show significant recovery with short-term (e.g., 1–5 weeks) and extended abstinence (e.g., 1–9 months) from alcohol (see Zou et al., 2018; Kirkland et al., 2022; Durazzo et al., 2024b and references therein). However, few studies have investigated how TMS affects regional brain volumes and neurometabolites following stimulation in those with AUD. In an open-label TMS study that administered 15 sessions of 20 Hz TMS (23,400 total pulses) over 5 days to the right DLPFC of individuals with AUD, no significant changes in regional cortical or subcortical volumes were observed post-treatment (Wu et al., 2018). In an RCT, Qiao et al. (2016) administered 20 sessions of active or sham 10 Hz TMS to the right DLPFC over 26 days and measured N-acetylaspartate (NAA; marker of neuronal integrity), choline-containing compounds (Cho; marker of cell membrane turnover/synthesis), and creatine-containing compounds (Cr; marker of cellular bioenergetics) (see Meyerhoff et al., 2013 for review of magnetic resonance-derived neurometabolites) in the bilateral hippocampi (NAA and Cho were scaled to Cr). The active TMS group, compared to sham, demonstrated significant bilateral increases in hippocampal NAA/Cr and Cho/Cr, suggesting that active TMS improved hippocampal neuronal integrity and cell membrane turnover/synthesis. However, no TMS RCT for AUD concurrently assessed for changes in brain volumes and neurometabolites in the stimulated brain region. Additionally, no published studies have specifically examined the effects of multiple intermittent theta burst sessions on brain structure and metabolites in individuals with AUD.

The goal of this study was to report longitudinal changes in brain volumes and neurometabolites in the left dorsolateral prefrontal cortex (DLPFC)—the stimulation site—across two RCTs evaluating intermittent theta burst stimulation (iTBS) as an adjunct treatment for AUD (see Padula et al., 2024; Durazzo et al., 2025 for primary outcomes). We predicted that participants who received active (active) versus sham (sham) iTBS, over the approximate 2-week iTBS intervention interval, demonstrate: (1) greater volume increases in the left rostral and caudal middle frontal and superior frontal gyri comprising the DLPFC; and (2) greater increases in left DLPFC NAA, Cho, Cr, gamma-aminobutyric acid (GABA), and glutamate (Glu). In the active group, we predicted that a greater number of left DLPFC iTBS pulses delivered at the target treatment level would be associated with larger increases in left rostral and caudal middle frontal and superior frontal gyri volumes and all aforementioned left DLPFC neurometabolites.

## 2 Methods and materials

### 2.1 Participants

**Figure 1:**
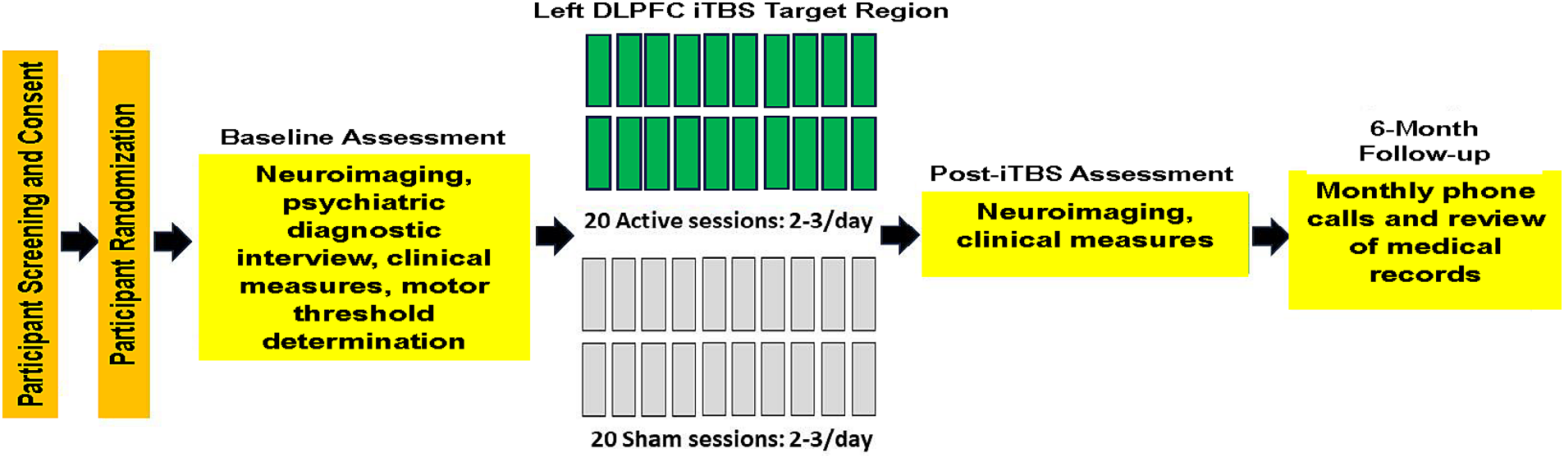
Study experimental timeline.

Veterans with AUD were recruited from a residential treatment program at the VA Palo Alto Health Care System (VAPAHCS). The treatment program duration was typically 28–35 days, and participants averaged 25 days of abstinence from alcohol before study enrollment. Participants in this report were in RCTs evaluating the efficacy of iTBS for promoting extended abstinence or significant reduction in alcohol consumption in Veterans in residential treatment for AUD (Padula et al., 2024, https://www.clinicaltrials.gov/search?id=NCT03291431 and Durazzo et al., 2025; NCT03191266, https://www.clinicaltrials.gov/search?id=NCT03191266). In Padula and colleagues, participants received 600 active or sham iTBS pulses per session, for 20 total sessions. In Durazzo et al., participants received 1,200 active or sham iTBS pulses per session, for 20 total sessions. See Figure 1 for the study experimental timeline for the foregoing RCTs. See Padula et al. (2024) and Durazzo et al. (2025) for CONSORT diagrams and study-specific participant demographic and clinical characteristics. Eleven participants were from the study by Padula et al. (2024) and 33 were from the study by Durazzo et al. (2025). Not all participants from our parent RCTs completed neuroimaging procedures due to extended scanner unavailability caused by scanner/facilities upgrades and COVID-19 restrictions; consequently, in this study, 21 participants were randomized to active iTBS (4 from Padula et al., 2024 and 17 from Durazzo et al., 2025) and 23 to sham iTBS (7 from Padula et al., 2024 and 16 from Durazzo et al., 2025). There were no differences between participants with and without neuroimaging on demographic, clinical, and alcohol consumption variables in the combined samples. All participants met the Diagnostic and Statistical Manual of Mental Disorders-5 (DSM-5) criteria for AUD, and 97% of the sample was classified with severe AUD. Participants provided written informed consent before the initiation of all study procedures. All procedures were approved by the VA Palo Alto HCS and Stanford University institutional review boards, and they observed the ethical standards outlined by the Declaration of Helsinki (see Table 1 for participant demographic and clinical characteristics). Study procedures were initiated on 06 December 2017 for Padula et al. (2024) and 16 April 2018 for Durazzo et al. (2025), with an approximate 3-year overlap in the recruiting period for these studies.

**Table 1:** Demographic and clinical measures.

Measure	Active (*n* = 21)	Sham (*n* = 23)	*p*-value
Age	48.2 (13.7)	49.3 (11.8)	0.75
Years of education	14.3 (2.0)	13.3 (1.3)	0.06
White (%)	71	78	0.73
Male (%)	100	96	0.99
Days abstinent at the initiation of study procedure	20 (13)	29 (30)	0.34
Days abstinent at conclusion of study procedures	35 (12)	44 (30)	0.36
Days in residential treatment (median)	29	29	0.99
Number of previous formal inpatient or outpatient treatment programs (median)	1	3	0.70
Baseline to post-assessment interval (days)	15 (3)	15 (3)	0.99
iTBS delivery interval	12 (3)	12 (3)	0.99
Number of DSM-5 alcohol use disorder criteria met (median)	10	11	0.67
Lifetime history of major depressive disorder (%)	52	48	0.99
PTSD, past month (%)	54	30	0.22
Substance use disorder (%)	19	22	0.99
Panic disorder, past month (%)	0	13	0.23
Obsessive-compulsive disorder, past month (%)	0	9	0.49
Beck Anxiety Inventory	13 (14)	15 (9)	0.64
Beck Depression Inventory-II	16 (11)	21 (11)	0.17
PTSD Checklist-5	55 (22)	59 (18)	0.47
Number of days drinking 3 months prior to the study (median)	66	67	0.88
Number of drinks 3 months prior to the study (median)	828	1,228	0.31
Drinks per drinking day 3 months prior to the study (median)	12	16	0.09
Cannabis Use Disorder Identification Test (median)	7	10	0.27
Smoking status (%)			All > 0.10
Never	19	34	
Former	52	22	
Current	29	44	
Anti-craving/anti-consumption (%)			All > 0.40
Disulfiram	0	0	
Acamprosate	5	4	
Topiramate	0	9	
Naltrexone	33	26	
Gabapentin	43	35	
Antidepressants (%)			All > 0.20
Selective serotonin reuptake inhibitor	38	17	
Serotonin-norepinephrine reuptake inhibitor	21	19	
Mirtazapine	10	0	
Bupropion	0	4	
Percent of participants predicted they received active treatment	86	83	0.99
Confidence in the rating of treatment assignment	7.0 (1.8)	7.2 (1.7)	0.50
Active motor threshold (median)	41	44	0.43
	Min = 34	Min = 32	
	Max = 55	Max = 55	
Resting motor threshold (median)	52	50	0.45
	Min = 37	Min = 42	
	Max = 65	Max = 76	
Number of pulses at target treatment level (median)	19,770	21,750	0.46
	Min = 3,870	Min = 9,480	
	Max = 23,850	Max = 23,850	

Mean and (standard deviation) unless otherwise indicated. Group comparisons on variables listed as mean and (standard deviation) were conducted with generalized linear model. Group comparisons on variables listed as percent were conducted with the Fisher’s Exact Test. Group comparisons on variables listed as medians were conducted with the Mann–Whitney Test.

### 2.2 Inclusion/exclusion criteria

Inclusion and exclusion criteria for the studies by Padula et al. (2024) and Durazzo et al. (2025) were identical. Primary inclusion criteria were adults aged 18 years and older, fluency and literacy in English, and enrollment in residential treatment for AUD at the start of the study procedures. Exclusion criteria included (1) presence of suicidal ideations representing imminent risk for self-harm; (2) clinically documented impairment of fine motor skills and auditory and/or visual acuity that would compromise performance in study procedures; (3) general medical conditions, diseases, or neurological disorders recognized to adversely affect neurocognition or brain neurobiology (i.e., cerebrovascular accident, multiple sclerosis, Alzheimer disease, Parkinson disease, space occupying cerebral lesion(s), etc.); (4) history of traumatic brain injury resulting in loss of consciousness greater than 10 min; and (5) current or past psychiatric diagnosis of bipolar, schizophrenia spectrum, and other psychotic disorders. The following comorbidities were allowed due to their high prevalence in AUD, particularly in Veterans: hypertension, hepatitis C, type-2 diabetes, unipolar mood disorders (major depression and substance-induced mood disorder), anxiety disorders (generalized anxiety disorder and panic disorder), and post-traumatic stress disorder (PTSD) (Durazzo and Meyerhoff, 2017; Nguyen et al., 2020; Durazzo et al., 2024a). Participants who met DSM-5 criteria for current or past substance use disorder were included, given the high prevalence of comorbid substance abuse in AUD (Dawson et al., 2005; Stinson et al., 2005; Mannes et al., 2021). Participants were urine-tested for illicit substances and breathalyzed for recent alcohol consumption before assessment. No participant tested positive for illicit or non-prescribed substances or had a detectable blood alcohol level at any assessment.

### 2.3 Psychiatric, substance, and drinking history assessment

At baseline (pre-iTBS), psychiatric diagnoses were assessed using the Mini-International Neuropsychiatric Interview for DSM-5 (MINI). Participants also completed the Clinical Interview for DSM-5 Alcohol Use Disorder and self-report questionnaires assessing demographics, medical history, and other substance use. The Timeline Follow-back (TLFB) obtained alcohol consumption over the 3 months before the study. Baseline depressive symptomatology and anxiety symptomatology were measured with the Beck Depression Inventory-II (BDI-II) and Beck Anxiety Inventory (BAI), respectively. PTSD symptomatology was assessed with the PTSD Checklist for DSM-5 (PCL-5). See Nguyen et al. (2020) for corresponding references for the above measures.

### 2.4 Magnetic resonance imaging (MRI) and magnetic resonance spectroscopy (MRS) acquisition

MRI and MRS data were acquired on a 3 T GE system (General Electric Healthcare, Milwaukee, WI, USA) equipped with a 32-channel head coil (Nova Medical, Wilmington, MA, USA) at the Stanford University Center for Cognitive and Neurobiological Imaging.

**Figure 2:**
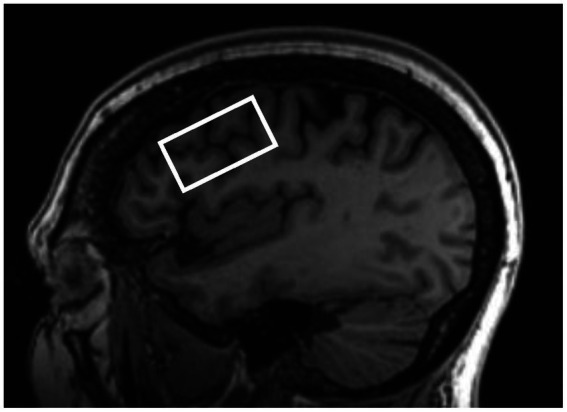
Representative left dorsolateral prefrontal cortex voxel placement.

T1-weighted images were acquired with the following parameters: repetition time (TR): 8.69 ms, echo time (TE): 3.44 ms, inversion time: 500 ms, 11-degree flip angle, 256 × 256 matrix, and 1 mm^3^ isotropic resolution. An improved MEGA-SPECIAL single voxel spectroscopy editing sequence (Gu et al., 2018) was used to obtain neurometabolite levels in the left DLPFC (editing ON/OFF = 1.9/7.5 ppm, TR/TE = 2,000/80 ms, 256 transients, and 10.6 min acquisition). The 40 × 22 × 22 mm^3^ (19.4 mL) single voxel was prescribed in the left dorsolateral prefrontal cortex localized from the 3D T1-weighted anatomical image using a semi-automated voxel placement procedure to place the voxel. This was accomplished by applying non-linear normalization to identify subject-specific MRI coordinates from a previously selected target coordinate located in the DLPFC from the Montreal Neurological Institute (MNI) template. During the semi-automated voxel placement procedure, after initial automated coordinate identification, the MRS voxel (Figure 2) was then aligned to the angle of the skull in the sagittal plane. Then, the left DLPFC mask in MNI standard space was co-registered to each participant’s T1-weighted image (Gozdas et al., 2022).

### 2.5 MRI and MRS processing

Regional brain volumes and intracranial volume (ICV) were quantified using FreeSurfer,^1^ via the v7.3.2 longitudinal pipeline (Reuter et al., 2012) from T1-weighted images. Images for baseline and post-assessment for each participant were first processed cross-sectionally, followed by rigorous visual inspection for parcellation/segmentation errors in FreeView. Manual editing, when required, was executed in FreeView,^2^ with criteria derived from our previous morphometric studies (Durazzo et al., 2011a, b). The most common manual edits were pial edits^3^ to eliminate dura artifact not fully removed by the skull stripping procedure. Only baseline and post-assessment images that passed QC standards for each participant were submitted to the longitudinal pipeline. Average volume, surface area, and thickness were generated for 34 bilateral cortical regions of interest (Desikan et al., 2006). In this study, we focus on bilateral volumes (mm^3^) of the rostral and caudal middle frontal and superior frontal gyri (*n* = 44). We included the right hemisphere volumes of the foregoing regions to serve as a reference for the predicted greater volume increases in the left hemisphere in active versus sham participants.

In the current study, we report on levels of NAA (combined concentrations of N-acetylaspartate and N-acetylaspartylglutamate, *n* = 41), total Cho (combined concentrations of phosphocholine and glycerophosphocholine, *n* = 41), and total Cr (combined concentrations of creatine and phosphocreatine, *n* = 41), Glu, and GABA. NAA, Cho, and Cr were quantified by fitting the editing OFF spectrum with LCModel [v 6.3-1 (Provencher, 2001)] and referenced to unsuppressed water. The GABA (*n* = 36) edited spectrum was obtained by subtracting the editing OFF spectrum from the editing ON spectrum. Glu (*n* = 36) was quantitated from the OFF spectrum using peak integration at 2.35 ppm. Water reference (i.e., area of the water peak) was estimated by integrating the water peak using the water-unsuppressed frames, acquired together with water-suppressed frames using the MEGA-SPECIAL sequence (Gu et al., 2018). All metabolites were scaled to water and reported in institutional units (i.u.). Gannet (v 3.3.2) was employed to quantify gray matter, white matter, and cerebrospinal fluid (CSF) fractions of the left DLPFC voxel (Edden et al., 2014). Across active and sham participants, average left DLPFC line width (Hz, FWHM), across baseline and post-assessment, was 9.0 ± 1.3. Over baseline and post-assessment, average Cramer-Rao lower bounds were 2.19 ± 0.61 for NAA, 2.91 ± 0.93 for Cho, and 3.04 ± 0.86 for Cr, which are well within recommended quality control standards for LC Model spectral fits for these metabolites (Provencher, 2001). Glu and GABA were quantified by peak integration (Gu et al., 2018); therefore, these metabolites do not have Cramer-Rao bounds equivalent to the LC Model. Across groups and assessment points, the mean and standard deviation for GABA were 9.13 and 2.80; the mean and standard deviation for Glu were 21.21 and 6.62. See Durazzo et al. (2023) for representative metabolite spectral fitting.

### 2.6 Statistical analyses

#### 2.6.1 Cross-sectional analyses

Active and sham groups were compared on baseline demographic and clinical variables via Fisher’s Exact Test, Mann–Whitney Test, or generalized linear model, as appropriate. Comparisons of active and sham groups on volumes and metabolites, at baseline and post-iTBS assessment, were conducted with generalized linear modeling and corresponding pairwise *t*-tests. Cohen’s *d* was calculated for all cross-sectional comparisons between active and sham on brain volumes and metabolites (Cohen, 1988). All statistical analyses were completed with SPSS v29.

#### 2.6.2 Longitudinal analyses

##### 2.6.2.1 Primary analyses

Longitudinal change in brain volumes and metabolites was evaluated with linear mixed modeling (LMM). Fixed variables included group assignment (active or sham) and specified binary and/or continuous covariates (see the Covariates section). Random intercepts were fit for continuous time (baseline to post-iTBS assessment interval), and final model parameters were estimated with restricted maximum likelihood estimation. The omnibus model for each dependent measure included main effects for group and time, group × time interaction, and covariates specified in the Covariates section; *p* < 0.05 was considered statistically significant for group and time and group × time interaction for brain volumes and metabolites.

##### 2.6.2.2 Preliminary simple effects tests

Given the modest sample sizes for the brain volumes and metabolites quantitated in this study, irrespective of findings for group, time, and group × time factors, simple effect tests were conducted to investigate changes in the above volume and metabolite measures within the active and sham participants. In the absence of significant group × time interactions, the simple effect tests were considered preliminary. LMM was used to compare the rate of change across hemispheres for the caudal and rostral middle frontal and superior frontal gyri within active and sham groups; we did not model the group × hemisphere × time second-order interaction because the limited number of participants would likely lead to overfitting the data. To control for multiplicity of tests for simple effects, we employed a modified Bonferroni procedure (see Sankoh et al., 1997), which adjusted simple effect test *p*-values for metabolites and volumes to account for the number of pairwise tests (five for metabolites and six for volumes) and the average Spearman intercorrelation among the five metabolites (r = 0.69) and six volumes (r = 0.51) across all participants at baseline; this procedure resulted in an adjusted alpha level of *p* ≤ 0.030 for metabolites and *p* ≤ 0.021 for bilateral volumes for simple effect tests for the active and sham groups. Standard Bonferroni multiplicity adjustment was not utilized as this procedure assumes orthogonality among dependent measures (see https://www.quantitativeskills.com/sisa/calculations/bonhlp.htm and Sankoh et al., 1997), which was not apparent with the neuroimaging measures acquired in this study, given the above intercorrelations. The multiplicity-corrected p-values for volumes and metabolites were applied to cross-sectional comparisons between active and sham participants. Cohen’s *d* for repeated measures (Lakens, 2013) was calculated for volumes and metabolites’ simple effects tests. Cohen’s *d* effect sizes for all cross-sectional and longitudinal analyses were interpreted as follows: small effect *d* < 0.50; medium effect *d* = 0.50–0.79, and large effect *d* ≥ 0.80 (Cohen, 1988).

##### 2.6.2.3 Exploratory analyses

In the active group, the number of pulses received at the target treatment level was used as a predictor of change for brain volumes and metabolites using LMM. In these analyses, volumes and metabolites served as the dependent measures. In the above exploratory analyses, *p* < 0.05 was considered statistically significant.

##### 2.6.2.4 Covariates

Study membership (binary variable coded for Padula et al., 2024 or Durazzo et al., 2025) was included as a covariate in all cross-sectional and longitudinal analyses. Age and ICV were included as covariates in all cross-sectional and longitudinal analyses comparing active and sham, due to their association with regional brain volumes in Veterans with AUD (Durazzo et al., 2015). Age and left DLPFC CSF fraction were included as covariates in all brain metabolite analyses because of their association with regional metabolites in Veterans with AUD (Mon et al., 2012). Additionally, in all cross-sectional and longitudinal comparisons of active and sham participants, the total number of alcohol-containing drinks 3 months before study or average number of drinks per drinking day 3 months before study served as covariates in final models to determine potential associations between alcohol consumption and change in volumes and metabolites. Gabapentin use was used as a covariate in all analyses for GABA and Glu (GABA is synthesized from Glu) because oral use may influence regional brain GABA levels (see Meyerhoff et al., 2018 and references therein). Finally, anti-craving and antidepressant medications use, as a binary class, and BDI-II score were individually included as covariates in the final models.

## 3 Results

### 3.1 Participants’ characteristics

Active and sham groups were not significantly different on demographic variables, frequency of psychiatric disorders or medications, smoking status, alcohol consumption variables, or BDI-II, BAI, or PCL-5 at baseline (see Table 1).

### 3.2 Cross-sectional volumes and metabolites

Active participants showed larger left superior frontal volume than sham at baseline [χ^2^(1) = 5.73, *p* = 0.017, Cohen’s *d* = 0.72]. At post-assessment, the active participants had higher GABA concentration than sham [χ^2^ (1) = 5.18, *p* = 0.023, Cohen’s *d* = 0.78]. Study membership, total number of alcohol-containing drinks 3 months before the study and average number of drinks per drinking day 3 months before the study were not significant predictors of volume in any region (all *p* > 0.25). Antidepressant and anti-craving medications (including specifically gabapentin) and BDI-II score were not associated with metabolite levels at baseline or post-assessment (all *p* > 0.15).

### 3.3 Longitudinal volumes

Main effects for time indicated significant increases for the left caudal middle frontal gyrus [*F*(1, 37) = 7.80, *p* = 0.008], left [*F*(1, 37) = 12.80, *p* < 0.001] and right rostral middle [*F*(1, 37) = 6.33, *p* = 0.016] frontal gyri, and left [*F*(1, 37) = 31.00, *p* < 0.001] and right superior frontal gyri [*F*(1, 37) = 13.20, *p* < 0.001]. A trend for increased right caudal middle frontal gyrus was observed [*F*(1, 37) = 1.92, *p* = 0.063]. There were no main effects for group or group × time interactions. Analysis comparing rates of change for left and right hemisphere caudal and rostral middle frontal and superior frontal gyri indicated no significant hemispheric differences in these volumes in the active or sham groups over the baseline-to-post-assessment interval (all *p* > 0.30). Study membership, total number of alcohol-containing drinks 3 months before the study and average number of drinks per drinking day 3 months before the study were not significant predictors of volume in any region (all *p* > 0.25). Antidepressant and anti-craving medications (including specifically gabapentin) and BDI-II score were not associated with change in any region (all *p* > 0.13).

### 3.4 Longitudinal metabolites

A main effect for time was yielded for Cho [*F*(1, 36) = 5.12, *p* = 0.030], with a trend for Cr [*F*(1, 36) = 4.07, *p* = 0.051], where both increased over the baseline-to-post-assessment interval. A group main effect was observed for GABA [*F*(1, 38) = 5.02, *p* = 0.031], where active participants had a higher GABA concentration over the 2-week assessment interval. There were no other main effects for group or group × time interactions. Study membership, total number of alcohol-containing drinks 3 months before the study, or average number of drinks per drinking day 3 months before the study were not significant predictors of any metabolite (all *p* > 0.20). Antidepressant and anti-craving medications (including specifically gabapentin) and BDI-II score were not associated with change in any metabolite (all *p* > 0.24).

### 3.5 Preliminary simple effects analyses

#### 3.5.1 Volume simple effect tests

**Figure 3:**
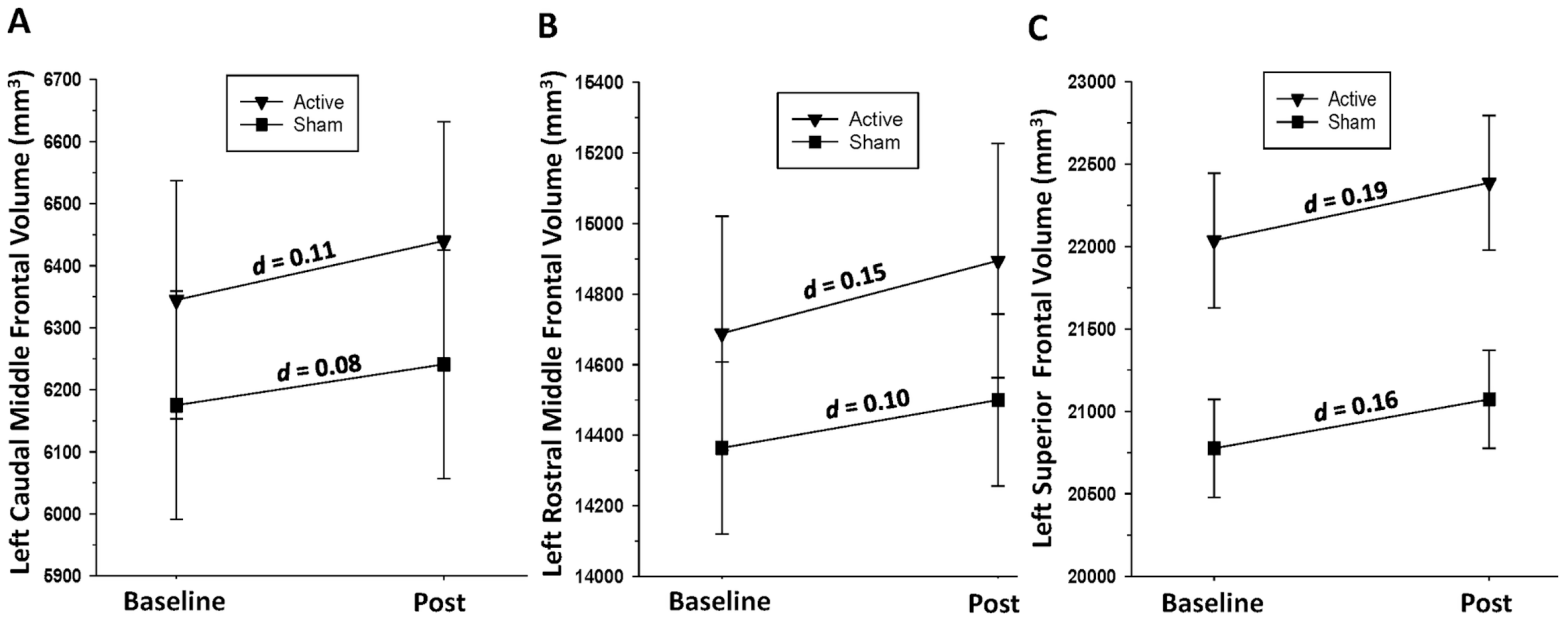
**(A)** Change in left caudal middle frontal gyrus volume over the baseline-to-post-assessment interval in active and sham groups. **(B)** Change in left rostral middle frontal gyrus volume over the baseline-to-post-assessment interval in active and sham groups. **(C)** Change in left superior frontal gyrus volume over the baseline-to-post-assessment interval in active and sham groups.

*Active group:* significant increases over the baseline-to-post-assessment interval were observed for the left [*F*(1, 17) = 18.79, *p* < 0.001] and right [*F*(1, 17) = 9.26, *p* = 0.007] caudal middle frontal gyri, left [*F*(1, 17) = 18.79, *p* < 0.001] and right [*F*(1, 17) = 15.71, *p* < 0.001] rostral middle frontal gyri, and left [*F*(1, 17) = 27.93, *p* < 0.001] and right [*F*(1, 17) = 15.65, *p* = 0.001] superior frontal gyri. *Sham group:* A significant increase over the baseline-to-post-assessment interval was observed for the left superior frontal gyrus [*F*(1, 19) = 8.52, *p* = 0.009] and a trend for the right superior frontal gyrus [*F*(1, 19) = 3.41, *p* = 0.08] (see Figure 3).

#### 3.5.2 Metabolite simple effect tests

**Figure 4:**
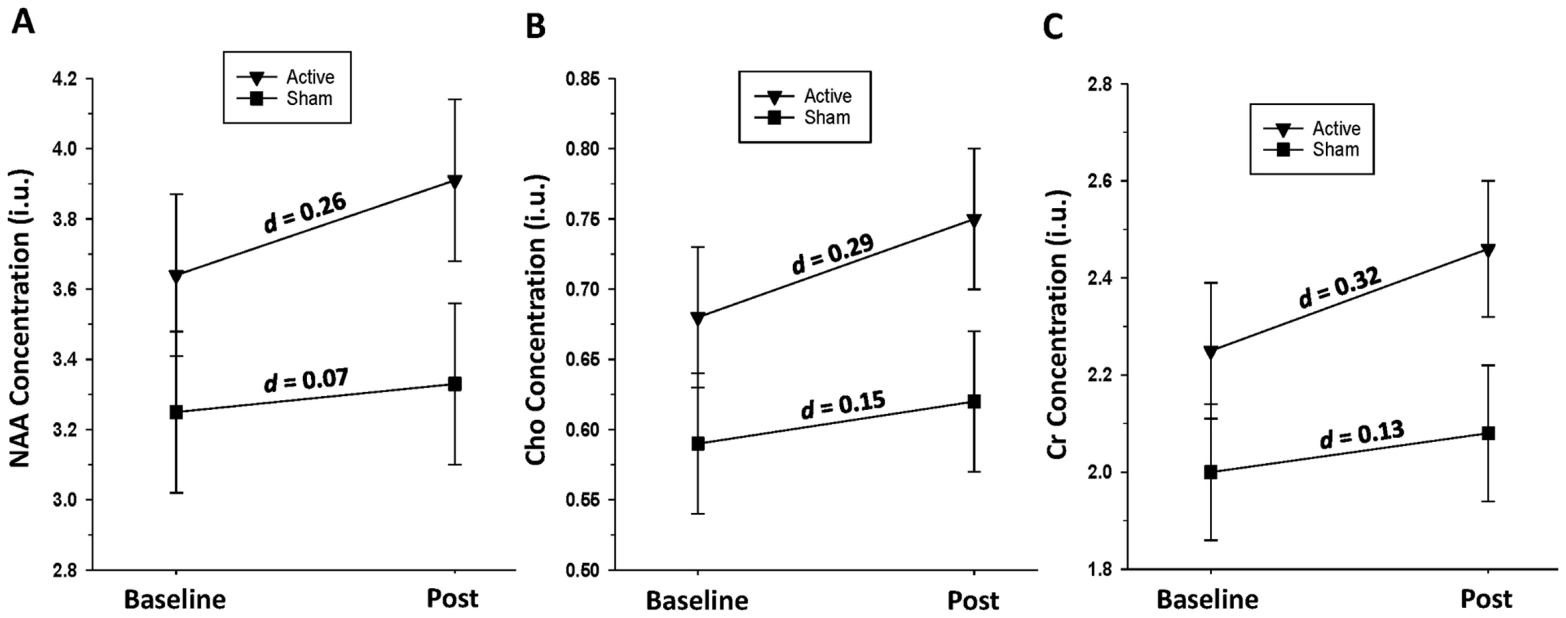
**(A)** Change in left dorsolateral prefrontal cortex n-acetylaspartate (NAA) level over the baseline-to-post-assessment interval in active and sham groups. **(B)** Change in left dorsolateral prefrontal cortex choline-containing compounds (Cho) level over the baseline-to-post-assessment interval in active and sham groups. **(C)** Change in left dorsolateral prefrontal cortex creatine-containing compounds (Cr) level over the baseline-to-post-assessment interval in active and sham groups.

*Active group:* Significant increases over the baseline-to-post-assessment interval were seen for Cho [*F*(1, 17) = 12.33, *p* = 0.003] and Cr [*F*(1, 17) = 6.20, *p* = 0.023], with trends for NAA [*F*(1, 17) = 4.23, *p* = 0.055] and Glu [*F*(1, 15) = 3.21, *p* = 0.093]. *Sham group:* No significant changes were observed in any metabolite over time (all *p* > 0.39) (see Figure 4).

### 3.6 Exploratory analyses—association between number of pulses at target treatment level and changes in metabolites and volumes in active participants

A higher number of pulses at the target treatment level was significantly associated with greater changes in NAA, Glu, and GABA (Table 2). There were no significant associations between the number of pulses at the target treatment level and change in volume for any region.

**Table 2:** Active participants associations between pulses delivered at the target treatment level and change in left DLPFC metabolite concentrations.

Metabolite	Slope of pulses at the target treatment	SE	*p*-value
NAA	8.61e-5	3.73e-5	0.033
Cho	1.24e-5	8.48e-6	0.161
Cr	4.62e-5	2.42e-5	0.072
Glu	2.40e-4	1.10e-4	0.032
GABA	5.40e-4	2.30e-4	0.038

SE, standard error of the estimate. See Section 2.6.2.3.

Exploratory analyses for analysis description.

## 4 Discussion

The main findings of this study are as follows: (1) preliminary analyses indicated active participants demonstrated volume increases in bilateral rostral and caudal middle frontal gyri and superior frontal gyri, while sham participants only demonstrated volume increases in the left superior frontal gyrus; (2) preliminary analyses indicated active participants had increases in Cho and Cr; sham participants showed no significant changes in neurometabolites; (3) exploratory analyses indicated that for active participants, a greater number of pulses received at target treatment level was associated with larger increases in NAA, Glu, and GABA concentrations.

Wu and colleagues reported no significant gray matter volume changes in any brain regions, subsequent to 23,400 pulses of 20 Hz stimulation, delivered to the right DLPFC, over approximately 5 days (no sham group for comparison), in those seeking treatment for AUD (Wu et al., 2018). The Authors suggested that the brief treatment duration may not have been sufficient to promote increased neural plasticity, as measured by structural neuroimaging. At the initiation of iTBS sessions, participants of the current study were abstinent from alcohol for approximately 3–4 weeks, a period associated with rapid increases in cortical volume (van Eijk et al., 2012; Durazzo et al., 2015; Zou et al., 2018) and thickness (Durazzo et al., 2024b), particularly in the bilateral DLPFC, in those with AUD undergoing typical inpatient or outpatient treatment. Both active and sham groups showed increases in bilateral caudal and rostral middle frontal and superior frontal gyri volumes, but these changes were only comparable across groups for the left superior frontal gyrus. In those with treatment-resistant MDD, Lan et al. (2016) reported significant volume increases in the anterior cingulate, left middle temporal gyrus, left insula, and right angular gyrus after 25 sessions of 10 Hz (75,000 total pulses) to the left DLPFC (no sham group). The volume increases were attributed to the potential increased release of neurotrophic factors. Preliminary analyses from the current study also indicated that active participants showed increased left DLPFC Cho, Cr levels, and a trend for NAA concentration, while sham had no metabolite changes. Qiao et al. (2016) found increased NAA/Cr and Cho/Cr in the bilateral hippocampi in those with AUD, after active right DLPFC 10 Hz stimulation, relative to sham participants. Metabolite changes in the right DLPFC were not investigated. Similar to brain morphology changes, the duration of alcohol abstinence in participants in the current study corresponds to a period associated with rapid increases in regional cortical and subcortical metabolites, particularly NAA and Cho, in individuals with AUD, especially in anterior frontal regions (Ende et al., 2005; Mon et al., 2012). The increased Cho and Cr levels, at the site of stimulation for active participants, suggest improved cell membrane turnover/synthesis and cellular bioenergetics in the large volume of tissue contained in the left DLPFC voxel.

Overall, volumetric findings for active participants suggest that iTBS bolstered volume recovery of the bilateral dorsolateral/dorsomedial cortex and Cho and Cr in the left DLPFC, over the brief 2-week administration interval. The bilateral volume increase in active participants is congruent with research indicating that iTBS, and other forms of TMS, can induce neuroplastic changes one or more synapses away from the site of stimulation (Jannati et al., 2023). Preclinical research on a single pulse train suggested an immediate glial cell response to iTBS, especially white matter plasticity indices (Ong and Tang, 2025), whereas multiple treatment sessions repolarized microglia and promoted neurogenesis through brain-derived neurotrophic factor (BDNF) in induced cerebrovascular accident models (Peipei et al., 2024). However, the only study, to the best of our knowledge, that investigated the effects of iTBS in a preclinical model of alcohol consumption found no change in cortical BDNF gene expression within 7 days of stimulation (Dhungana et al., 2025). The interval from stimulation to biomarker measurement, as well as the brain region stimulated, in preclinical models and humans, may significantly influence the interpretation of how TMS promotes changes in brain neurobiology (Ong and Tang, 2025). Therefore, the mechanism(s) promoting the preliminary indications of greater volumetric and metabolite increases of active participants in this study are unclear and warrant further investigation, specifically for glial proliferation and the release of growth factors, as potential contributing pathways. The volume and metabolite gains observed in active participants did not result in significant differences between groups at post-assessment (except for GABA), which suggests that the assessment interval was potentially too short and/or the sample was underpowered to detect plasticity-related group divergence in the regions investigated. Despite the preliminary nature of the volumetric and neurometabolite changes in active participants, the overall findings suggest that iTBS produced adaptive neuroplastic changes in the left DLPFC and homologous regions of the right hemisphere. The volumetric and metabolite changes observed in active participants suggest improved integrity of tissue of the left DLPFC, a central cortical node of the executive function network; improved integrity of left DLPFC tissue may relate to enhanced regulation/control of affect and goal-directed behavior in the context of previously experienced alcohol-related stimuli and psychosocial stressors (Durazzo et al., 2011b).

Human and preclinical research suggests that structural and neurometabolite recovery, in those with AUD during early and extended abstinence, is likely related to increases in neuronal dendritic arbor, soma/cell volume, synaptogenesis/synaptic density, glial proliferation (particularly astrocytes and microglia), and remyelination (Dlugos and Pentney, 1997; Crews et al., 2004; Sullivan and Pfefferbaum, 2005; Crews and Nixon, 2009; Miguel-Hidalgo, 2018; Fritz et al., 2019). The neuronal components (e.g., dendrites/dendritic spines and cell bodies) and glial cells (e.g., protoplasmic astrocytes) that combine to form the parenchyma of cortex may recover at different rates in many regions during early versus extended abstinence from alcohol (Durazzo et al., 2015; Zou et al., 2018; Durazzo et al., 2024b). Therefore, the timing of TMS treatment initiation, as well as the treatment interval, may influence the efficacy of this intervention for AUD.

In active participants, the association of a higher number of pulses received at target treatment level with larger increases in NAA, Glu, and GABA concentrations, after adjusting for study membership, suggests a potential dose–response relationship between a modifiable iTBS parameter and neuronal integrity and the general metabolic pool of Glu and GABA that corresponds to left DLPFC region stimulated. Specifically, it may be advisable to monitor the number of trains administered at the target treatment level during each session and add target-level trains to compensate for any pulses delivered below the target treatment level during ramping procedures (Durazzo et al., 2025).

The modest sample size of predominantly male Veteran composition limits the generalizability of these findings. We fully acknowledge that there were no group × time interactions for the longitudinal brain volumes and metabolite measures of this study. We consider the findings from the simple effect tests conducted to be preliminary to guide future larger-scale mechanistic studies on iTBS treatment response in humans. Additionally, assessment of other regions that form nodes of the executive, salience, and mood regulation circuits, in conjunction with the iTBS protocol of our iTBS studies, may inform modification of iTBS and other treatment protocols to optimize adaptive long-term post-treatment functioning, including sustained abstinence. Future iTBS studies for AUD should incorporate a larger proportion of females, considering large-scale TMS RCTs reported that biological sex may influence clinical outcomes in depressive disorders (Kedzior et al., 2014; Sackeim et al., 2020). Premorbid factors (e.g., genetic risk or resiliency factors) and comorbid factors (e.g., diet/nutrition, exercise, and subclinical hepatic, pulmonary, cardiac, or cerebrovascular dysfunction) that were not assessed in this study may have influenced the cross-sectional and longitudinal findings.

In conclusion, this study provided novel preliminary indications that iTBS promoted adaptive structural and neurometabolite changes in the left DLPFC stimulation site in Veterans in residential treatment for AUD. Replication of these findings and exploration of other neuroimaging-based markers of TMS-induced neurobiological changes are critical to informing modifications of existing TMS protocols to maximize enduring positive treatment outcomes.

## Data Availability

The raw data supporting the conclusions of this article will be made available by the authors, without undue reservation.

## Footnotes

^1^ https://surfer.nmr.mgh.harvard.edu/fswiki

^2^ https://freesurfer.net/fswiki/Edits

^3^ https://surfer.nmr.mgh.harvard.edu/fswiki/FsTutorial/PialEdits_freeview

## References

[ref1] Antonelli M. Fattore L. Sestito L. Di Giuda D. Diana M. Addolorato G. (2021). Transcranial magnetic stimulation: a review about its efficacy in the treatment of alcohol, tobacco and cocaine addiction. Addict. Behav. 114:106760. doi: 10.1016/j.addbeh.2020.106760, PMID: 33316590

[ref2] Beynel L. Powers J. P. Appelbaum L. G. (2020). Effects of repetitive transcranial magnetic stimulation on resting-state connectivity: a systematic review. NeuroImage 211:116596. doi: 10.1016/j.neuroimage.2020.116596, PMID: 32014552 PMC7571509

[ref3] Cohen J. (1988). Statistical power analysis for the behavioral sciences. Hillsdale, NJ: Lawrence Erlbaum Associates.

[ref4] Cole E. O'Sullivan S. J. Tik M. Williams N. R. (2024). Accelerated theta burst stimulation: safety, efficacy, and future advancements. Biol. Psychiatry 95, 523–535. doi: 10.1016/j.biopsych.2023.12.004, PMID: 38383091 PMC10952126

[ref5] Crews F. T. Collins M. A. Dlugos C. Littleton J. Wilkins L. Neafsey E. J.. (2004). Alcohol-induced neurodegeneration: when, where and why? Alcohol. Clin. Exp. Res. 28, 350–364. doi: 10.1097/01.ALC.0000113416.65546.01, PMID: 15112943

[ref6] Crews F. T. Nixon K. (2009). Mechanisms of neurodegeneration and regeneration in alcoholism. Alcohol Alcohol. 44, 115–127. doi: 10.1093/alcalc/agn079 18940959 PMC2948812

[ref7] Dawson D. A. Grant B. F. Stinson F. S. Chou P. S. Huang B. Ruan W. J. (2005). Recovery from DSM-IV alcohol dependence: United States, 2001-2002. Addiction 100, 281–292. doi: 10.1111/j.1360-0443.2004.00964.x 15733237

[ref8] Desikan R. S. Segonne F. Fischl B. Quinn B. T. Dickerson B. C. Blacker D.. (2006). An automated labeling system for subdividing the human cerebral cortex on MRI scans into gyral based regions of interest. NeuroImage 31, 968–980. doi: 10.1016/j.neuroimage.2006.01.021, PMID: 16530430

[ref90001] Dhungana A. McCalley D. Heath A. Kraybill E. Mojabi F. Morales J.. (2025). Developing a reverse translational model of low-intensity rTMS in alcohol use disorder: the influence of theta burst stimulation protocols on binge alcohol drinking in mice. Transcranial Magnetic Stimulation, 4. doi: 10.1016/j.transm.2025.100098, PMID: 40585525 PMC12201979

[ref9] Dlugos C. A. Pentney R. J. (1997). Morphometric evidence that the total number of synapses on Purkinje neurons of old F344 rats is reduced after long-term ethanol treatment and restored to control levels after recovery. Alcohol Alcohol. 32, 161–172. doi: 10.1093/oxfordjournals.alcalc.a008250, PMID: 9105510

[ref10] Durazzo T. C. Kraybill E. P. Stephens L. H. May A. C. Meyerhoff D. J. (2024a). Pro-atherogenic medical conditions are associated with widespread regional brain metabolite abnormalities in those with alcohol use disorder. Alcohol Alcohol. 59. doi: 10.1093/alcalc/agae055, PMID: 39127890 PMC11316785

[ref11] Durazzo T. C. Kraybill E. P. Stephens L. H. McCalley D. M. Humphreys K. May A. C.. (2025). Intermittent theta burst to the left dorsolateral prefrontal cortex promoted decreased alcohol consumption and improved outcomes in those with alcohol use disorder: a randomized, double-blind, placebo-controlled clinical trial. Drug Alcohol Depend. 270:112641. doi: 10.1016/j.drugalcdep.2025.112641, PMID: 40048832 PMC13064820

[ref9001] Durazzo T. C. McNerney M. W. Hansen A. M. Gu M. Sacchet M. D. Padula C. B. (2023). BDNF rs6265 Met carriers with alcohol use disorder show greater age-related decline of N-acetylaspartate in left dorsolateral prefrontal cortex. Drug Alcohol Depend, 248, 109901. doi: 10.1016/j.drugalcdep.2023.109901 37146499

[ref12] Durazzo T. C. Meyerhoff D. J. (2017). Psychiatric, demographic, and brain morphological predictors of relapse after treatment for an alcohol use disorder. Alcohol. Clin. Exp. Res. 41, 107–116. doi: 10.1111/acer.13267, PMID: 27883214 PMC6193554

[ref9002] Durazzo T. C. Mon A. Gazdzinski S. Meyerhoff D. J. (2011a). Chronic cigarette smoking in alcohol dependence: associations with cortical thickness and N-acetylaspartate levels in the extended brain reward system. Addict Biol, 18, 379–91. doi: 10.1111/j.1369-1600.2011.00407.x 22070867 PMC4157587

[ref13] Durazzo T. C. Mon A. Gazdzinski S. Yeh P. H. Meyerhoff D. J. (2015). Serial longitudinal magnetic resonance imaging data indicate non-linear regional gray matter volume recovery in abstinent alcohol-dependent individuals. Addict. Biol. 20, 956–967. doi: 10.1111/adb.12180, PMID: 25170881 PMC4345147

[ref14] Durazzo T. C. Stephens L. H. Meyerhoff D. J. (2024b). Regional cortical thickness recovery with extended abstinence after treatment in those with alcohol use disorder. Alcohol 114, 51–60. doi: 10.1016/j.alcohol.2023.08.011, PMID: 37657667 PMC10902196

[ref9003] Durazzo T. C. Tosun D. Buckley S. Gazdzinski S. Mon A. Fryer S. L. (2011b). Cortical thickness, surface area, and volume of the brain reward system in alcohol dependence: relationships to relapse and extended abstinence. Alcohol Clin Exp Res, 35, 1187–200. doi: 10.1111/j.1530-0277.2011.01452.x 21410483 PMC3097308

[ref15] Edden R. A. Puts N. A. Harris A. D. Barker P. B. Evans C. J. (2014). Gannet: a batch-processing tool for the quantitative analysis of gamma-aminobutyric acid–edited MR spectroscopy spectra. J. Magn. Reson. Imaging 40, 1445–1452. doi: 10.1002/jmri.24478, PMID: 25548816 PMC4280680

[ref16] Ende G. Welzel H. Walter S. Weber-Fahr W. Diehl A. Hermann D.. (2005). Monitoring the effects of chronic alcohol consumption and abstinence on brain metabolism: a longitudinal proton magnetic resonance spectroscopy study. Biol. Psychiatry 58, 974–980. doi: 10.1016/j.biopsych.2005.05.038 16084857

[ref17] Fox M. D. Halko M. A. Eldaief M. C. Pascual-Leone A. (2012). Measuring and manipulating brain connectivity with resting state functional connectivity magnetic resonance imaging (fcMRI) and transcranial magnetic stimulation (TMS). NeuroImage 62, 2232–2243. doi: 10.1016/j.neuroimage.2012.03.035, PMID: 22465297 PMC3518426

[ref18] Fritz M. Klawonn A. M. Zahr N. M. (2019). Neuroimaging in alcohol use disorder: from mouse to man. J. Neurosci. Res. 100, 1140–1158. doi: 10.1002/jnr.24423, PMID: 31006907 PMC6810809

[ref19] George M. S. Bohning D. E. Lorberbaum J. P. Nahas Z. Anderson B. Borckardt J. J.. (2007). “Overview of transcranial magnetic stimulation: history mechanisms physics and safety” in Transcranial magnetic stimulation in clinical psychiatry. eds. George M. S. Belmaker R. H. (Washington, DC; London, England: American Psychiatric Publishing, Inc), 1–38.

[ref20] Gozdas E. Hinkley L. Fingerhut H. Dacorro L. Gu M. Sacchet M. D.. (2022). (1)H-MRS neurometabolites and associations with neurite microstructures and cognitive functions in amnestic mild cognitive impairment. Neuroimage Clin. 36:103159. doi: 10.1016/j.nicl.2022.103159, PMID: 36063758 PMC9450331

[ref21] Gu M. Hurd R. Noeske R. Baltusis L. Hancock R. Sacchet M. D.. (2018). GABA editing with macromolecule suppression using an improved MEGA-SPECIAL sequence. Magn. Reson. Med. 79, 41–47. doi: 10.1002/mrm.26691, PMID: 28370458 PMC5623603

[ref22] Harel M. Perini I. Kämpe R. Alyagon U. Shalev H. Besser I.. (2022). Repetitive transcranial magnetic stimulation in alcohol dependence: a randomized, double-blind, sham-controlled proof-of-concept trial targeting the medial prefrontal and anterior cingulate cortices. Biol. Psychiatry 91, 1061–1069. doi: 10.1016/j.biopsych.2021.11.020, PMID: 35067356

[ref23] Jannati A. Oberman L. M. Rotenberg A. Pascual-Leone A. (2023). Assessing the mechanisms of brain plasticity by transcranial magnetic stimulation. Neuropsychopharmacology 48, 191–208. doi: 10.1038/s41386-022-01453-8, PMID: 36198876 PMC9700722

[ref24] Jansen J. M. van Wingen G. van den Brink W. Goudriaan A. E. (2015). Resting state connectivity in alcohol dependent patients and the effect of repetitive transcranial magnetic stimulation. Eur. Neuropsychopharmacol. 25, 2230–2239. doi: 10.1016/j.euroneuro.2015.09.019, PMID: 26481907

[ref25] Kedzior K. K. Azorina V. Reitz S. K. (2014). More female patients and fewer stimuli per session are associated with the short-term antidepressant properties of repetitive transcranial magnetic stimulation (rTMS): a meta-analysis of 54 sham-controlled studies published between 1997-2013. Neuropsychiatr. Dis. Treat. 10, 727–756. doi: 10.2147/ndt.S58405, PMID: 24855360 PMC4019615

[ref26] Kirkland A. E. Browning B. D. Green R. Leggio L. Meyerhoff D. J. Squeglia L. M. (2022). Brain metabolite alterations related to alcohol use: a meta-analysis of proton magnetic resonance spectroscopy studies. Mol. Psychiatry 27, 3223–3236. doi: 10.1038/s41380-022-01594-8, PMID: 35508628 PMC10578135

[ref27] Kirkovski M. Donaldson P. H. Do M. Speranza B. E. Albein-Urios N. Oberman L. M.. (2023). A systematic review of the neurobiological effects of theta-burst stimulation (TBS) as measured using functional magnetic resonance imaging (fMRI). Brain Struct. Funct. 228, 717–749. doi: 10.1007/s00429-023-02634-x, PMID: 37072625 PMC10113132

[ref28] Lakens D. (2013). Calculating and reporting effect sizes to facilitate cumulative science: a practical primer for t-tests and ANOVAs. Front. Psychol. 4:863. doi: 10.3389/fpsyg.2013.00863, PMID: 24324449 PMC3840331

[ref29] Lan M. J. Chhetry B. T. Liston C. Mann J. J. Dubin M. (2016). Transcranial magnetic stimulation of left dorsolateral prefrontal cortex induces brain morphological changes in regions associated with a treatment resistant major depressive episode: an exploratory analysis. Brain Stimul. 9, 577–583. doi: 10.1016/j.brs.2016.02.011, PMID: 27017072 PMC5554068

[ref30] Long F. Chen Y. Zhang Q. Li Q. Wang Y. Wang Y.. (2024). Predicting treatment outcomes in major depressive disorder using brain magnetic resonance imaging: a meta-analysis. Mol. Psychiatry 30, 825–837. doi: 10.1038/s41380-024-02710-6, PMID: 39187625

[ref31] Mannes Z. L. Shmulewitz D. Livne O. Stohl M. Hasin D. S. (2021). Correlates of mild, moderate, and severe alcohol use disorder among adults with problem substance use: validity implications for DSM-5. Alcohol. Clin. Exp. Res. 45, 2118–2129. doi: 10.1111/acer.14701, PMID: 34581461 PMC8602758

[ref32] Mehta D. D. Praecht A. Ward H. B. Sanches M. Sorkhou M. Tang V. M.. (2024). A systematic review and meta-analysis of neuromodulation therapies for substance use disorders. Neuropsychopharmacology 49, 649–680. doi: 10.1038/s41386-023-01776-0, PMID: 38086901 PMC10876556

[ref33] Meyerhoff D. J. Durazzo T. C. Ende G. (2013). Chronic alcohol consumption, abstinence and relapse: brain proton magnetic resonance spectroscopy studies in animals and humans. Curr. Top. Behav. Neurosci. 13, 511–540. doi: 10.1007/7854_2011_131 21688208

[ref34] Meyerhoff D. J. Murray D. E. Durazzo T. C. Pennington D. L. (2018). Brain GABA and glutamate concentrations following chronic gabapentin administration: a convenience sample studied during early abstinence from alcohol. Front. Psych. 9:78. doi: 10.3389/fpsyt.2018.00078, PMID: 29599727 PMC5862797

[ref35] Miguel-Hidalgo J. J. (2018). Molecular neuropathology of astrocytes and oligodendrocytes in alcohol use disorders. Front. Mol. Neurosci. 11:78. doi: 10.3389/fnmol.2018.00078, PMID: 29615864 PMC5869926

[ref36] Mon A. Durazzo T. Meyerhoff D. J. (2012). Glutamate, GABA, and other cortical metabolite concentrations during early abstinence from alcohol and their associations with neurocognitive changes. Drug Alcohol Depend. 125, 27–36. doi: 10.1016/j.drugalcdep.2012.03.012, PMID: 22503310 PMC3419314

[ref37] Nguyen L. C. Durazzo T. C. Dwyer C. L. Rauch A. A. Humphreys K. Williams L. M.. (2020). Predicting relapse after alcohol use disorder treatment in a high-risk cohort: the roles of anhedonia and smoking. J. Psychiatr. Res. 126, 1–7. doi: 10.1016/j.jpsychires.2020.04.003, PMID: 32403028 PMC8476113

[ref38] Ong R. C. S. Tang A. D. (2025). Subthreshold repetitive transcranial magnetic stimulation induces cortical layer-, brain region-, and protocol-dependent neural plasticity. Sci. Adv. 11:eado6705. doi: 10.1126/sciadv.ado6705, PMID: 39772671 PMC11708880

[ref39] Padula C. B. McCalley D. M. Tenekedjieva L. T. MacNiven K. Rauch A. Morales J. M.. (2024). A pilot, randomized clinical trial: left dorsolateral prefrontal cortex intermittent theta burst stimulation improves treatment outcomes in veterans with alcohol use disorder. Alcohol Clin. Exp. Res. (Hoboken) 48, 164–177. doi: 10.1111/acer.15224, PMID: 38197808

[ref40] Padula C. B. Tenekedjieva L. T. McCalley D. M. Al-Dasouqi H. Hanlon C. A. Williams L. M.. (2022). Targeting the salience network: a Mini-review on a novel Neuromodulation approach for treating alcohol use disorder. Front. Psych. 13:893833. doi: 10.3389/fpsyt.2022.893833, PMID: 35656355 PMC9152026

[ref41] Peipei W. Yu D. Xiaoyan L. Yunxia L. Liuming L. Tongbin C.. (2024). Effects of a novel regimen of repetitive transcranial magnetic stimulation (rTMS) on neural remodeling and motor function in adult male mice with ischemic stroke. J. Neurosci. Res. 102:e25358. doi: 10.1002/jnr.25358, PMID: 38859672

[ref42] Philip N. S. Sorensen D. O. McCalley D. M. Hanlon C. A. (2020). Non-invasive brain stimulation for alcohol use disorders: state of the art and future directions. Neurotherapeutics 17, 116–126. doi: 10.1007/s13311-019-00780-x, PMID: 31452080 PMC7007491

[ref43] Provencher S. W. (2001). Automatic quantitation of localized *in vivo* ^1^H spectra with LC model. NMR Biomed. 14, 260–264. doi: 10.1002/nbm.698, PMID: 11410943

[ref44] Qiao J. Jin G. Lei L. Wang L. Du Y. Wang X. (2016). The positive effects of high-frequency right dorsolateral prefrontal cortex repetitive transcranial magnetic stimulation on memory, correlated with increases in brain metabolites detected by proton magnetic resonance spectroscopy in recently detoxified alcohol-dependent patients. Neuropsychiatr. Dis. Treat. 12, 2273–2278. doi: 10.2147/ndt.S106266, PMID: 27695332 PMC5028171

[ref45] Reuter M. Schmansky N. J. Rosas H. D. Fischl B. (2012). Within-subject template estimation for unbiased longitudinal image analysis. NeuroImage 61, 1402–1418. doi: 10.1016/j.neuroimage.2012.02.084, PMID: 22430496 PMC3389460

[ref46] Sackeim H. A. Aaronson S. T. Carpenter L. L. Hutton T. M. Mina M. Pages K.. (2020). Clinical outcomes in a large registry of patients with major depressive disorder treated with transcranial magnetic stimulation. J. Affect. Disord. 277, 65–74. doi: 10.1016/j.jad.2020.08.005, PMID: 32799106

[ref47] Sankoh A. J. Huque M. F. Dubey S. D. (1997). Some comments on frequently used multiple endpoint adjustment methods in clinical trials. Stat. Med. 16, 2529–2542. doi: 10.1002/(SICI)1097-0258(19971130)16:22<2529::AID-SIM692>3.0.CO;2-J 9403954

[ref48] Stinson F. S. Grant B. F. Dawson D. A. Ruan W. J. Huang B. Saha T. (2005). Comorbidity between DSM-IV alcohol and specific drug use disorders in the United States: results from the National Epidemiologic Survey on alcohol and related conditions. Drug Alcohol Depend. 80, 105–116. doi: 10.1016/j.drugalcdep.2005.03.009, PMID: 16157233

[ref49] Sullivan E. V. Pfefferbaum A. (2005). Neurocircuitry in alcoholism: a substrate of disruption and repair. Psychopharmacology 180, 583–594. doi: 10.1007/s00213-005-2267-6, PMID: 15834536

[ref50] van Eijk J. Demirakca T. Frischknecht U. Hermann D. Mann K. Ende G. (2012). Rapid partial regeneration of brain volume during the first 14 days of abstinence from alcohol. Alcohol. Clin. Exp. Res. 37, 67–74. doi: 10.1111/j.1530-0277.2012.01853.x, PMID: 23072363

[ref51] Wu G. R. Baeken C. Van Schuerbeek P. De Mey J. Bi M. Herremans S. C. (2018). Accelerated repetitive transcranial magnetic stimulation does not influence grey matter volumes in regions related to alcohol relapse: an open-label exploratory study. Drug Alcohol Depend. 191, 210–214. doi: 10.1016/j.drugalcdep.2018.07.004, PMID: 30142603

[ref52] Zou X. Durazzo T. C. Meyerhoff D. J. (2018). Regional brain volume changes in alcohol-dependent individuals during short-term and Long-term abstinence. Alcohol. Clin. Exp. Res. 42, 1062–1072. doi: 10.1111/acer.13757, PMID: 29672876 PMC5984169

